# *Trypanosoma cruzi* Epimastigotes Are Able to Manage Internal Cholesterol Levels under Nutritional Lipid Stress Conditions

**DOI:** 10.1371/journal.pone.0128949

**Published:** 2015-06-11

**Authors:** Miria Gomes Pereira, Gonzalo Visbal, Leonardo T. Salgado, Juliana Cunha Vidal, Joseane L. P. Godinho, Nuccia N. T. De Cicco, Geórgia C. Atella, Wanderley de Souza, Narcisa Cunha-e-Silva

**Affiliations:** 1 Laboratório de Ultraestrutura Celular Hertha Meyer, Instituto de Biofísica Carlos Chagas Filho, Universidade Federal do Rio de Janeiro, Rio de Janeiro, Brasil; 2 Instituto Nacional de Metrologia, Qualidade e Tecnologia—INMETRO, Rio de Janeiro, Brasil; 3 Instituto de Pesquisas Jardim Botânico do Rio de Janeiro, Rio de Janeiro, Brasil; 4 Laboratório de Bioquímica de Lipídios e Lipoproteínas, Instituto de Bioquímica Médica Leopoldo de Meis, Universidade Federal do Rio de Janeiro, Rio de Janeiro, Brasil; Universidade Federal de Juiz de Fora, BRAZIL

## Abstract

*Trypanosoma cruzi* epimastigotes store high amounts of cholesterol and cholesteryl esters in reservosomes. These unique organelles are responsible for cellular digestion by providing substrates for homeostasis and parasite differentiation. Here we demonstrate that under nutritional lipid stress, epimastigotes preferentially mobilized reservosome lipid stocks, instead of lipid bodies, leading to the consumption of parasite cholesterol reservoirs and production of ergosterol. Starved epimastigotes acquired more LDL-NBD-cholesterol by endocytosis and distributed the exogenous cholesterol to their membranes faster than control parasites. Moreover, the parasites were able to manage internal cholesterol levels, alternating between consumption and accumulation. With normal lipid availability, parasites esterified cholesterol exhibiting an ACAT-like activity that was sensitive to Avasimibe in a dose-dependent manner. This result also implies that exogenous cholesterol has a role in lipid reservoirs in epimastigotes.

## Introduction

*Trypanosoma cruzi*, the causative agent of Chagas disease, which affects more than 8 million of people in the Latin American continent [1], belongs to a group of organisms that does not synthesise cholesterol, producing instead mainly ergosterol. Many of these organisms, including protists and fungi, are human parasites. This remarkable difference in sterol metabolism has encouraged researchers to search for sterol biosynthesis inhibitors that act specifically on the divergent steps between cholesterol and ergosterol pathways [2, 3]

Despite being unable to produce cholesterol, *T*. *cruzi* does acquire it from medium, as demonstrated by the uptake and accumulation of LDL-gold particles in lysosome-like organelles, called reservosomes [4]. Moreover, there is a remarkable accumulation of cholesterol and cholesteryl esters in crystalloid structures inside reservosomes that the parasites are able to mobilise [5].

Reservosomes are implicated in storing nutrients to be consumed during metacyclogenesis [6]. Remarkably, proteomic analysis of isolated reservosomes [7] identified a set of enzymes involved in lipid metabolism, namely sterol-24-methyltransferase, sterol-24-reductase, NADPH reductase (related to ergosterol production), lipases, and a homologue of Rab18, a small GTPase involved in the release of lipids from lipid bodies in mammals. The ABCA1 transporter, associated with cholesterol efflux in human cells, was also found; it had already been immunolocalised in *T*. *cruzi* reservosomes [8], although its function was not clarified. These data suggest that reservosome play a pivotal role in parasite metabolism, which includes endogenous and exogenous lipid management.

The parasite also present many uncharacterised lipid bodies distributed throughout the cytoplasm, mainly in the trypomastigote form. In this way, the parasite can coordinate two different lipid stocks according to cellular demand. In this work, we addressed the mobilisation of cholesterol from the stocks in reservosomes and/or lipid bodies during periods of lipid starvation, as well as the ability to insert the exogenous cholesterol into the parasite’s membranes. Moreover, we also investigated if exogenous cholesterol could be esterified, suggesting an ACAT-related enzyme activity in *T*. *cruzi*.

## Material and Methods

### Parasites

*T*. *cruzi* epimastigotes (Y strain) were cultivated for 3–4 days at 28°C in LIT (liver-infusion tryptose) medium [9] supplemented with 10% foetal calf serum (FCS) (Vitrocell, São Paulo, Brazil) or 10% delipidated FCS (dFCS). Cell density was measured by direct counting in a Neubauer chamber.

### Foetal calf serum delipidation

Lipid extraction of FCS (Vitrocell, São Paulo, Brazil) without protein precipitation was performed as described [10].

### Electron Microscopy

Epimastigotes cultivated in LIT medium supplemented with FCS or dFCS were washed in phosphate buffered saline (PBS, 150 mM NaCl in 10 mM sodium phosphate buffer, pH 7.2), fixed in 2.5% glutaraldehyde and 4% formaldehyde in 100 mM sodium phosphate buffer, pH 7.2, for 60 min at room temperature, post fixed in 1% osmium tetroxide, 0.8% potassium ferrocyanide, 5 mM calcium chloride in 100 mM cacodylate buffer, pH 7.2, for 60 min, dehydrated in an acetone series and embedded in Epoxy resin. Ultrathin sections were stained with 5% uranyl acetate and lead citrate and observed with a Zeiss 900 transmission electron microscope operating at 80 kV. To perform morphometric analysis, cells in random sections were registered until 30 different cells from each experimental situation were analysed. The acquired images were processed with ImageJ software (NIH, Bethesda, USA). The number of pixels was adjusted to the correspondent micrometer length at the micrograph scale (1 μm equivalent to 436 pixels, at magnification of 30,000X).

### Lipid analysis

Parasites (1 x 10^8^ cells) were cultivated in LIT supplemented with 10% dFCS for 0, 24, 48 and 72 h at 28°C and washed in PBS. Lipids were extracted using the Folch method as follows [11]: 12 mL of extraction solution (chloroform-methanol, 2:1, vol/vol) was added to a glass test tube containing 2 mL of sample to yield a final ratio of chloroform/methanol/water (4:2:1). The mixture was stirred vigorously using a vortex machine, and the suspension was kept at 5°C for five days. To produce a biphasic layer, the suspension was poured into a separating funnel followed by 40 mL of chloroform and 10 mL of brine and shaken briefly. Then the lower organic phase was drained, and the extraction procedure was repeated one more time with 15 mL of chloroform. The organic extract was dried, concentrated and suspended with 5 mL of chloroform. The extract was divided into two equal aliquots. One aliquot was applied to a silicic acid column (1.5 cm × 4 cm) [12–14] and the neutral lipids were eluted with 5 column volumes of chloroform and collected as a single fraction. The solvent was evaporated in a rotary evaporator under vacuum at 50°C, and the neutral lipids were suspended and transferred into a 10 mL conical centrifuge tube where the sample was dried under nitrogen to be subsequently analyzed by gas chromatography (GC) with mass Spectrometric detection (GC-MS).

The other aliquot was poured into a glass test tube for the saponification process [15]. Briefly, after concentrating the organic extract under nitrogen, 1 mL of methanolic base (1.0 M potassium hydroxide in methanol freshly prepared) was added and saponification carried out for 30 min at 80°C in a water bath. At the end of specified hydrolysis time, 1 mL of 1.0 M hydrochloride acid was added to neutralize, followed by 200 μL brine and 2 mL ethyl acetate to extract the unsaponifiable portion. The organic phase was transferred into a 10 mL glass test tube with a pipette and dried under nitrogen. The organic extract was suspended with 2 mL of chloroform and applied to a silicic acid column (1.5 cm × 4 cm), washed with 5 column volumes of chloroform to separate neutral lipids from other lipid as described earlier.

### Free sterols analysis

For quantitative analysis and structural assignment, neutral lipids were separated in a capillary high-resolution column (30 m x 0.25 mmi.d.HP-5MS column, (5% diphenyl 95% dimethyl polysilxane, 0.25 μm film thickness) in a Agilent Technologies 7890A gas chromatographer equipped with mass-sensitive detector Agilent Technologies 5975C inert XL MSD. Lipids were dissolved in ethyl acetate and injected into the column at an initial temperature of 50°C (1 min), followed by a temperature increase to 270°C at a rate of 20°C/min and further raise to 300°C at a rate of 1°C/min. The carrier gas (He) flow was kept constant at 1 mL/min. Injector temperature was 250°C; the detector was kept at 280°C.

### Calibration for Cholesterol and Ergosterol Determination

A set of five calibration standards was prepared from the pure standard of cholesterol and ergosterol purchased from Sigma-Aldrich Co. Different calibration solutions were prepared using ethyl acetate as solvent. For the quantification of cholesterol and ergosterol, standards were used at different concentrations of 0.01, 0.04, 0.10, 0.30, 0.50 and 1.0 mM to plot the standard curve. From each calibration solution, 1 μL was injected (run in triplicate) into the GC-MS system to achieve the regression plot of various concentrations versus their peak area.

### Lipoprotein purification

Low density lipoprotein (LDL) was purified from fresh human plasma as described previously [16] and modified by our group [17].

### Preparation of NBD-cholesterol-labelled LDL

Purified LDL was labelled with NBD-cholesterol (22-(N-(7-nitrobenz-2-oxa-1,3-diazol-4-yl) amino)-23,24-bisnor-5-cholen-3ß-ol) (Molecular Probes—Invitrogen, Carlsbad, CA) as described by [18] with slight modifications. Briefly, 1 mg of NBD-cholesterol dissolved in ethanol was dried under nitrogen stream and mixed with 10 mL of fresh human plasma. After incubation for 24 hours at 37°C, the LDL-NBD-cholesterol particles were purified as described for LDL particles. LDL labelling was confirmed by UV exposition of samples previously separated by electrophoresis. Protein quantification was performed using a DC Protein Assay (Bio-Rad) using albumin as the standard.

### Preparation of ^3^H-cholesterol-labelled LDL

^3^H-CHO-LDL was obtained as described by [17]. ^3^H-cholesterol (PerkinElmer, Boston, MA, USA) stored in chloroform-methanol (2:1) was dried under nitrogen stream, resolubilized in 30 μL of absolute ethanol and mixed with 12 mL of fresh human plasma. After incubation for 24 hours at 37°C, the LDL particles were purified as described above, and the specific radioactivity was determined by liquid scintillation counting. Protein quantification was performed using the DC Protein Assay (Bio-Rad) using albumin as the standard.

### Endocytosis assays

#### LDL—NBD-cholesterol endocytosis and image analysis

Parasites (2 x10^7^ cells) were incubated in LIT medium supplemented with 10% FCS (control) or 10% dFCS for 16–18 h at 28°C to induce parasite lipid starvation. After, epimastigotes were washed in serum-free medium and incubated in 400 μL LDL-NBD-Cholesterol in LIT to a final concentration of 1 mg/mL for 30 min at 28°C. Parasites were then washed and resuspended in LIT supplemented with 10% FCS for additional 0, 0.5, 1 and 2 h. Aliquots from each time point were collected, washed in PBS and fixed in 4% formaldehyde in PBS for 20 min at room temperature. Cell suspensions were adhered to 0.1% poly-L-lysine coated glass coverslips for 20 min and mounted with Prolong Gold with DAPI (Life Technologies, Grand Island, NY, USA). Parasites were observed using a Leica TCS SPE AOBS confocal laser scanning microscope (mounted on a DMI4000B inverted microscope), equipped with a 63X oil immersion objective (APO NA = 1.3). NBD-Chol and DAPI fluorescences were obtained with 488 nm and 405 nm excitation wavelengths, respectively. For all observations, the same acquisition parameters were used as follows: 1) an emission wavelength interval, corresponding to NBD-cholesterol fluorescence, ranging from 510 nm to 580 nm; 2) an emission wavelength interval, corresponding to DAPI fluorescence, ranging from 450 nm to 490 nm; 3) an image resolution of 2048 x 2048 pixels; 4) a pinhole setting at 137.2 μm (airy 1); 5) both laser powers standardised; 6) standardised photomultiplier adjustments (gain and offset); 7) the same step size (0.21 μm) and voxel volume (124906.528 nm^3^) for each image sequence obtained. For the quantification of NBD-cholesterol fluorescence in reservosomes/vesicles and plasma membranes, the background was first subtracted from a 3-dimensional stack using the 3D deconvolution tool of Leica LAS AF software. Fluorimetric analysis was performed using the same software to obtain the values of pixel intensity per area (mean grey value of pixel sum per pixel count). The number of cells analysed from two independent experiments were as follows: starved parasites (time zero, *n* = 25 cells; 0.5 h, *n* = 25 cells; 1 h, *n* = 22 cells; 2 h, *n* = 16 cells) and control parasites (time zero, *n* = 26 cells; 0.5 h, *n* = 18 cells; 1 h, *n* = 24 cells; 2 h, *n* = 15 cells).

#### LDL—Incorporation of 3H-cholesterol into cholesteryl esters

Aiming to determine the parasite ability of uptake and metabolise exogenous cholesterol in esters, parasites (1 x10^8^cells in 10 mL) were incubated in LIT medium supplemented with 10% FCS (control) or 10% dFCS (starved) for 16–18 h at 28°C before the addition of LDL– ^3^H-cholesterol (1,000,00 DPM, 2.0 mg/mL) and incubation for more 3 days. Cell suspensions were washed twice in PBS and lipid extractions were performed according to De Cicco *et al*. [17]. The lipid extracts were analysed by one-dimensional thin-layer chromatography (TLC) on Silica Gel 60 plates (E. Merck, Darmstadt, Germany) for neutral lipids using n-hexane:diethyl ether:acetic acid (60:40:1 v/v) [19]. Cholesterol, cholesteryl-oleate, glycerol-tryoleate, diolein, oleoyl-glycerol and oleic acid (Sigma-Aldrich Co, USA) were used as standards. The lipids were visualised using iodine vapour. The cholesterol and cholesteryl ester spots were isolated from the silica gel and re-extracted, using methanol:chloroform (2:1 v/v). The associated radioactivity was measured by liquid scintillation in a TRI-CARB 2810-TR (Perkin Elmer).

#### Determination of Acyl-CoA:cholesterol-acyltransferase activity by the incorporation of palmitic acid

Cholesterol esterification activity was assessed by treating the parasites with Sandoz 58–035–58–035 or Avasimibe (Sigma-Aldrich, St. Louis, MO, USA). Parasites (1 x 10^7^ cells/mL) were washed in LIT medium, resuspended in LIT plus 10% FCS and supplemented with Sandoz 58–035–58–035 in the concentrations of 0, 20, 30 μg/mL for 16 h or Avasimibe at 0, 5, 10 and 20 μM of palmitic acid for 24 h at 28°C. After drug incubations, 800,000 DPM of ^3^H-palmitic acid (palmitic acid [9, 10-^3^H (N)], PerkinElmer, Boston, MA, USA) complexed with free-fatty-acid-BSA was added for an additional 24 h. The cells were subjected to lipid extraction, and the lipids were analysed by thin-layer chromatography and re-extraction as described above. Cholesteryl-ester-associated radioactivity was measured by liquid scintillation counting.

The effect of drugs on the viability of *T*. *cruzi* epimastigotes was determined by the 3-(4,5-dimethylthiazol-2-yl)-2,5-diphenyl tetrazolium bromide (MTS) assay [20]. Briefly, the cells were collected, transferred into 24-well plates (approximately 3 x 10^6^ cells/well) and then incubated for 40 or 48 h in the presence of various concentrations of Sandoz 58–035 and Avasimibe, respectively. After incubation, washed cells were suspended in 100 μl of PBS in 96-well plates. Twenty microlitres of MTS/PMS solution was added to each well followed by incubation for 4 h. The reaction was read as optical density at 490 nm in a microplate spectrofluorometer SpectraMax M2/M2e (Molecular Devices). Additionally, treated parasites’ plasma membrane permeability was assessed by incubation in 20 μg/mL of propidium iodide for 15 min and reading in a BD Accury flow cytometer [21].

## Results and Discussion

### Epimastigotes consumed reservosome lipid content before the cytoplasmic stocks

**Fig 1:**
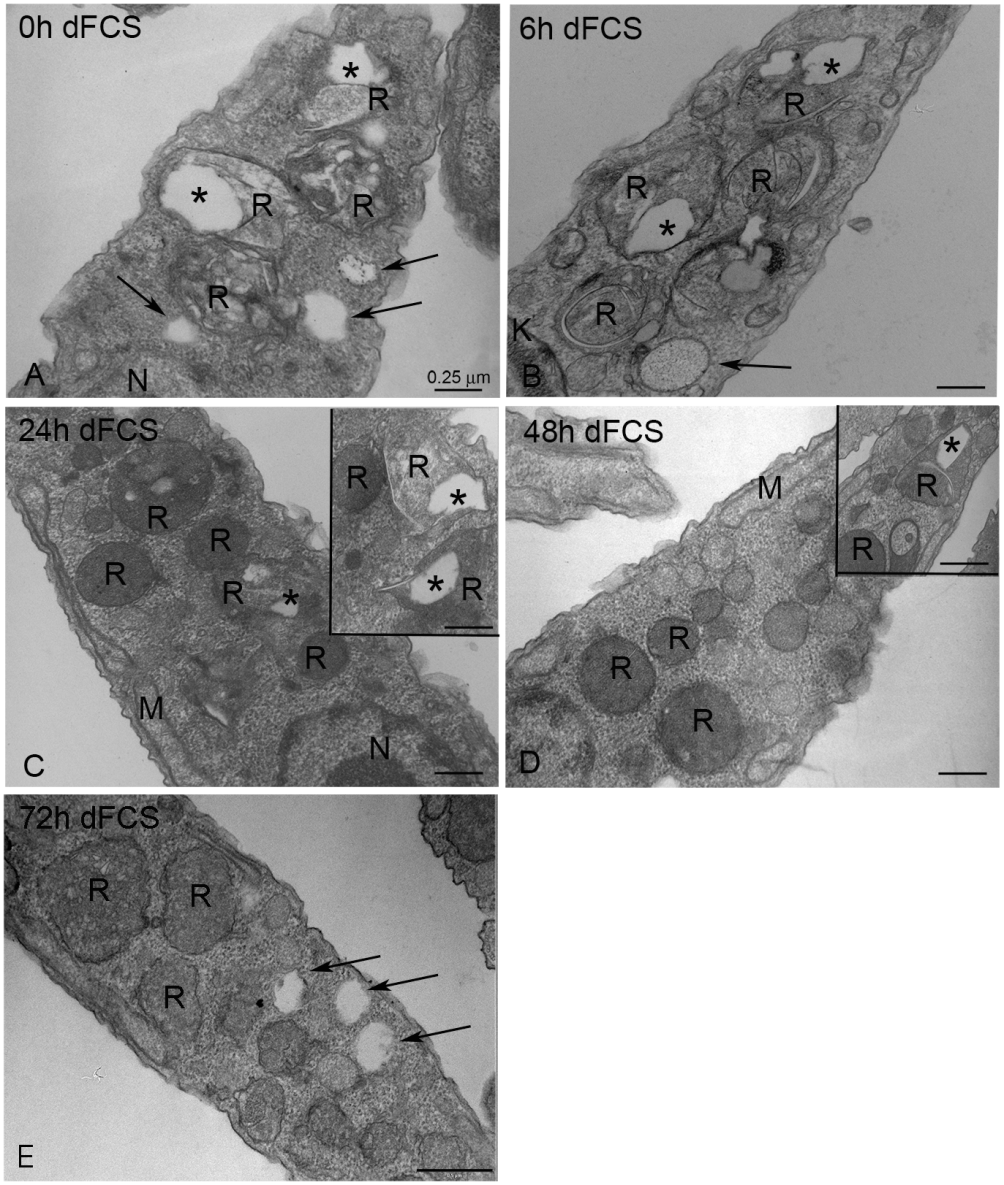
Transmission electron microscopy of epimastigotes cultivated in LIT medium supplemented with 10% dFCS. (A) 0 h—Reservosomes show many lipid inclusions in their lumen (asterisk). (B) After 6 h of lipid starvation, reservosomes filled with lipid inclusions could still be observed, but after 24 h (C), 48 h (D) and 72 h (E), an expressive decrease in reservosome lipids was registered. The insets show that there were reservosomes with typical lipid inclusions even after 24 to 72 h of starvation. The arrows point to lipid bodies throughout the cytoplasm. K- Kinetoplast, M—Mitochondrion, N—Nucleus, R- reservosomes. Bars correspond to 0.25 μm.

In order to analyse how epimastigotes manage their lipid stocks when submitted to lipid starvation, we cultivated the parasites in LIT medium supplemented with 10% dFCS at 28°C, taking aliquots at 0, 6 h, 24 h, 48 h and 72 h and processing for transmission electron microscopy (Fig 1). We chose electron microscopy to perform this analysis because it allows for the clear distinction between reservosome lipid inclusions and cytoplasmic lipid bodies. Previous work using ultrastructural cytochemistry and lipid analysis of purified fractions [5] have shown that the reservosome inclusions that appear electron lucent without any special processing are indeed composed of neutral lipids, such as cholesteryl esters and cholesterol. Before lipid starvation (time zero, Fig 1A) and after 6 h (Fig 1B), both the reservosome inclusions and the cytoplasmic lipid bodies were still the same size, but after 24 h reservosomes gradually lost their lipid inclusions (Fig 1C–1E). Table 1 shows the quantification of this reduction by morphometric analysis of the ultrathin sections. Notably, there was a reduction of approximately 72% of reservosome lipid inclusion area after 72 h of nutritional stress. Measurement of total reservosome area showed that the organelle did not undergo the gradual involution described during metacyclogenesis induced by nutritional stress [6]. Even after 72 h, parasites did not exhaust their reservosome lipids. A possible explanation is that the residual lipids supplied by liver infusion medium are able to sustain the parasites’ minimum lipid demands.

**Table 1:** Morphometric analysis of the area of reservosomes and the area occupied by lipid inclusions inside them in epimastigotes cultivated in LIT medium supplemented with 10% dFCS.

Parasites	Mean area of reservosomes (μm^2^) ^1^	Mean area of lipid inclusions per reservosome (μm^2^)^2^	Percentage of lipid inclusion reduction
Time zero	0.26669 ± 0.0116	0.03995 ± 0.0003	--
6 h dFCS	0.25955 ± 0.0111	0.04308 ± 0.0008	--
24 h dFCS	0.27268 ± 0.0186	0.01870 ± 0.0003*	53.20%
48 h dFCS	0.26239 ± 0.0141	0.01124 ± 0.0005*	71.87%
72 h dFCS	0.27745 ± 0.0207	0.01117 ± 0.0002*	72.05%

^1^ n = 30 parasite profiles;

^2^ n = 50 reservosomes

*P < 0.05 in relation to time zero. The results are expressed as the mean ± S.E.M using one-way ANOVA

We also analysed the number and area occupied by cytoplasmic lipid bodies in the parasites under the same starvation conditions (Table 2). The results revealed that neither the lipid body number nor their area varied, indicating that in situations of lipid starvation, the reservosomes’ lipid stock was consumed before the cytoplasmic lipid bodies. In a previous work, we already demonstrated the consumption of 70% of the reservosome lipid inclusions after 48 h in dFCS [5], but cytoplasmic lipid bodies had not been investigated. Our present data are consistent with the observations of epimastigote reservosome content consumption during metacyclogenesis accompanied by the increase in trypomastigote lipid bodies [6]. It is important to note that our experimental conditions did not engage epimastigotes in metacyclogenesis, indicating that exogenous neutral lipids are not the key nutrients whose depletion may trigger the process.

**Table 2:** Morphometric analysis of the number and the area occupied by lipid bodies in the cytoplasm of epimastigotes cultivated in LIT medium supplemented with 10% dFCS.

Parasites	Mean number of lipid bodies^1^	Mean area of lipid bodies (μm^2^)*
Time zero	2.8 ± 0.2932	0.04783 ± 0.00281
6 h dFCS	2.4 ± 0.3308	0.04144 ± 0.00226
24 h dFCS	3.1 ± 0.4921	0.04576 ± 0.00417
48 h dFCS	1.8 ± 0.3147	0.04404 ± 0.00231
72 h dFCS	3.2 ± 0.4482	0.04885 ± 0.02506

^1^ n = 30 parasite profiles

*P > 0.05. The results are expressed as the mean ± S.E.M using one-way ANOVA.

### Exogenous lipid starvation leads to cholesterol reservoirs consumption

Aiming to evaluate the changes in sterol profile after lipid starvation, we analysed by gas chromatography and mass spectrometry (GC-MS) the free and esterified sterol extracted from epimastigotes cultivated in LIT medium supplemented with 10% dFCS for 0, 24, 48 and 72 h. Free sterol composition was the expected for *T*. *cruzi* epimastigotes (Table 3). However, the cholesterol proportion diminished with time, representing a reduction of 37.2% in free cholesterol content in epimastigotes. While the C-24 alkylated sterols showed a slight increase in their relative mass to compensate the cholesterol reduction. Free ergosterol rate was maintained.

**Table 3:** Free sterols present in *T*. *cruzi* epimastigotes kept in LIT supplemented with dFCS for 0, 24, 48 and 72h.

Compounds/Molecular structure	RT	Relative ratio			
	(min)	0	24h	48h	72h
Cholesterol	23.2	37.1	35.1	28.2	23.3
Ergosterol	25.2	20.2	12.3	18.9	19.6
24-methyl-cholesta-5,22-dien-3β-ol	26.7	------	------	------	13.7
Ergosta-5,7-drien-3β-ol	26.8	7.1	------	4.7	----
24-ethyl-cholesta-5,7,22-trien-3β-ol	27.8	15.1	14.4	16.7	17.2
Sitosterol	28.4	13.6	25.4	21.2	18.1
24-ethyl-cholesta-7,24(24’)-drien-3β-ol	28.9	6.9	12.8	10.3	8.1

The sterol compositions were determined by GC-MS analysis as described in Materials and Methods.

Total sterols in starved parasites were analysed by GC-MS after saponification (Table 4). Cholesterol represents more than 87% of sterol composition before starvation. Moreover, the cholesterol proportion diminishes gradually (from 87.9 to 52.2%) according to starvation time and it is compensated by ergosterol rise (from 6.6 to 18.8%) and 24-ethyl-cholesta-5,7,22-trien-3β-ol (from 5.5 to 15.7%). These results indicate that esterified cholesterol is reduced to free cholesterol whereas ergosterol and 24-ethyl-cholesta-5,7,22-trien-3β-ol are metabolized to esters, although in lower proportion when compared to cholesteryl ester content.

**Table 4:** Total sterols present in *T*. *cruzi* epimastigotes kept in LIT supplemented with 10% dFCS for 0, 24, 48 and 72h.

Compounds/Molecular structure	RT	Relative ratio			
	(min)	0h	24h	48h	72h
Cholesterol		87.9	76.6	71.9	57.2
Ergosterol	25.2	6.6	11.8	14.9	18.8
Ergosta-5,7-drien-3β-ol	23.2	------	------	------	8.3
24-ethyl-cholesta-5,7,22-trien-3β-ol	25.2	5.5	11.6	13.2	15.7
	26.8				
	27.8				

Parasites were analysed by GC-MS after saponification as described in Materials and Methods.

Table 5 shows the free and total amount of cholesterol and ergosterol analysis in epimastigotes submitted to lipid stress. According to our results, total cholesterol mass is higher than free cholesterol. Before and after the first 24h of starvation in dFCS, the saponified samples presented masses of 187 and 144 μg of cholesterol, respectively. When we compare the same lipid stress times in non-saponified samples, the amount of free cholesterol was 27 and 23 μg, respectively, which represents a ratio of 6:1 of total to free cholesterol. The proportion falls to 2:1 after 48h and 72h of lipid starvation. Together, these results reveal that cholesteryl esters from reservosomes and/or lipid bodies probably begin to produce free cholesterol when parasites are maintained in medium with dFCS. Since we did not observe significant variation in lipid bodies’ number or area by electron microscopy, we conclude that most of sterol stocks that were consumed came from reservosomes. Moreover, the ergosterol analysis showed that free ergosterol mass increased, but the total ergosterol content was kept around 15 μg for 48h, reaching 20 μg after 72h. At that same time of starvation, the free ergosterol was higher than esterified ergosterol species, suggesting that part of esterified ergosterol might have been hydrolyzed to yield free ergosterol and to partially sustain parasite demand.

**Table 5:** Free and total (after saponification) cholesterol and ergosterol contents in lipid starved *T*. *cruzi* epimastigotes, kept in LIT medium supplemented with 10% dFCS for 0, 24, 48 and 72h.

Sample in dFCS	Free cholesterol (μg)	Total cholesterol (μg)	Free ergosterol (μg)	Total ergosterol (μg)
0h	27.0	187.0	7	14
24h	23.0	144.0	5	16
48h	40.0	73.0	11	14
72h	49.0	107.0	16	20

All samples correspond to 5 μg/mL of protein.

These results emphasise that in situations of nutritional lipid stress, parasites preferentially consume the cholesterol and cholesteryl esters previously stored instead of endogenous ergosterol.

It is notable that, in the opposite situation, the effects of ergosterol synthesis inhibition in *T*. *cruzi* was not reversed by the addition of exogenous cholesterol [22], suggesting that in this parasite form, the destination and function of the two sterol molecules are indeed independent. In *T*. *brucei* and *Leishmania*, the metabolism of cholesterol and ergosterol seems to be linked. The procyclic forms of *T*. *brucei* synthesise ergosterol and are able to uptake LDL particles by cysteine-rich acidic transmembrane protein (CRAM) receptors that function as LDL receptors in these parasites [18]. The high uptake of cholesterol by LDL leads to the down regulation of HMG-CoA reductase [23, 24]. The authors assumed that parasites keep an equilibrium between endogenous production (ergosterol) and cholesterol uptake from medium. In *Leishmania amazonensis*, parasites uptake cholesterol by LDL endocytosis via lipid raft mechanisms [17], and inhibitors of sterol biosynthesis increase LDL uptake by *L*. *amazonensis* [25], suggesting that the exogenous lipids, essential for parasite viability and proliferation, represent a compensatory mechanism to endogenous synthesis inhibition.

### Epimastigotes insert the exogenous cholesterol into their membranes

**Fig 2:**
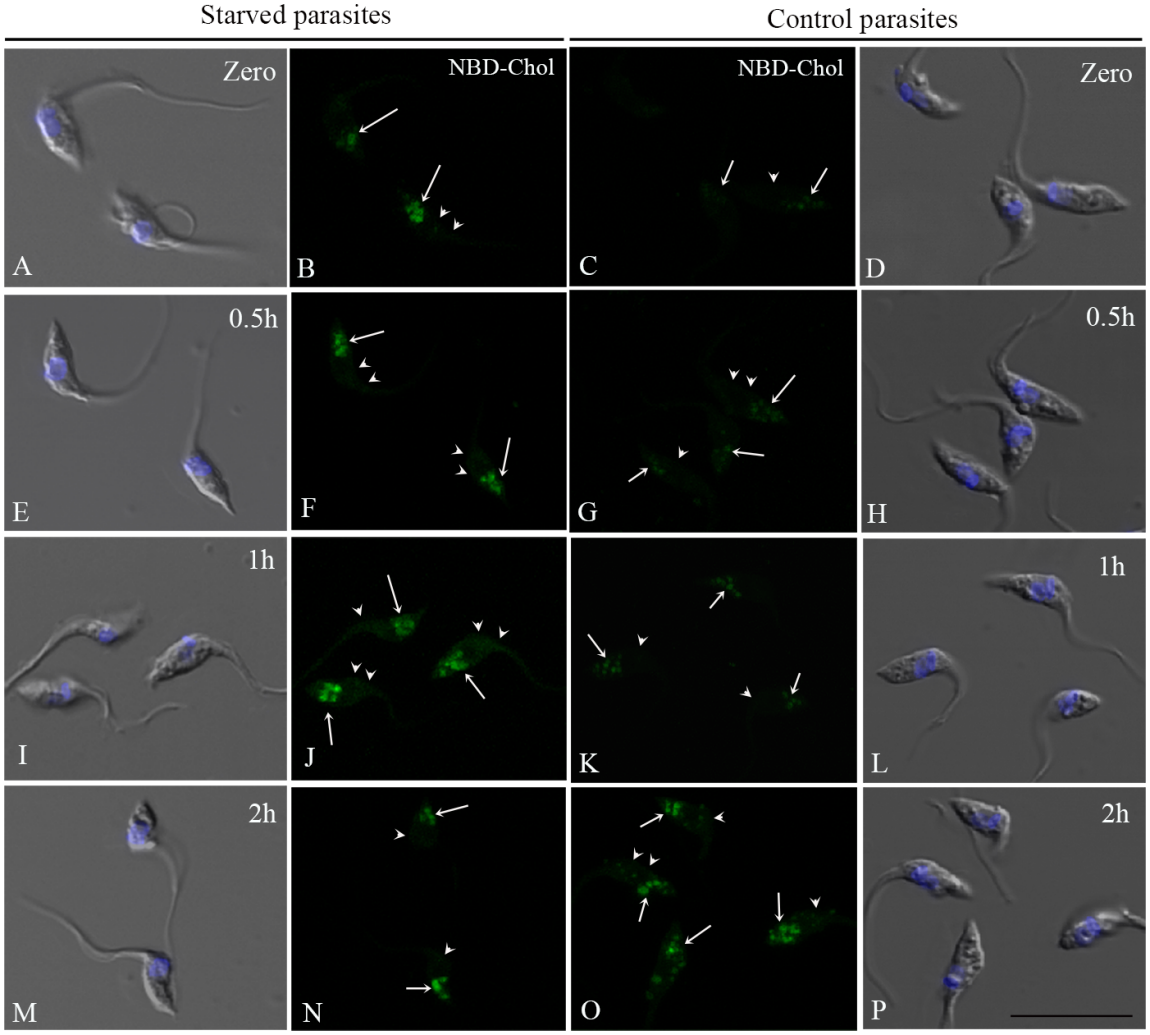
Distribution of NBD-cholesterol in *T*. *cruzi* epimastigotes. Starved and control parasites were incubated with LDL-NBD-cholesterol (1 mg/mL) for 30 min at 28°C, to concentrate the tracer in reservosomes, washed, resuspended in LIT + 10% FCS and chased for 0 h (A-D), 0.5 h (E-H), 1 h (I-L) and 2 h (M-P) for the fluorescent tracer distribution. Figures B, C, F, G, J, K, N and O are NBD fluorescence of formaldehyde fixed parasites, all registered under the same conditions. Figures A, D, E, H, I, L, M and P are overlays of DIC images and DAPI staining, used to label the nucleus and kinetoplast positions. The white arrows point to reservosomes and the arrowheads to the NBD-cholesterol in parasite membrane. Bar: 10 μm.

**Fig 3:**
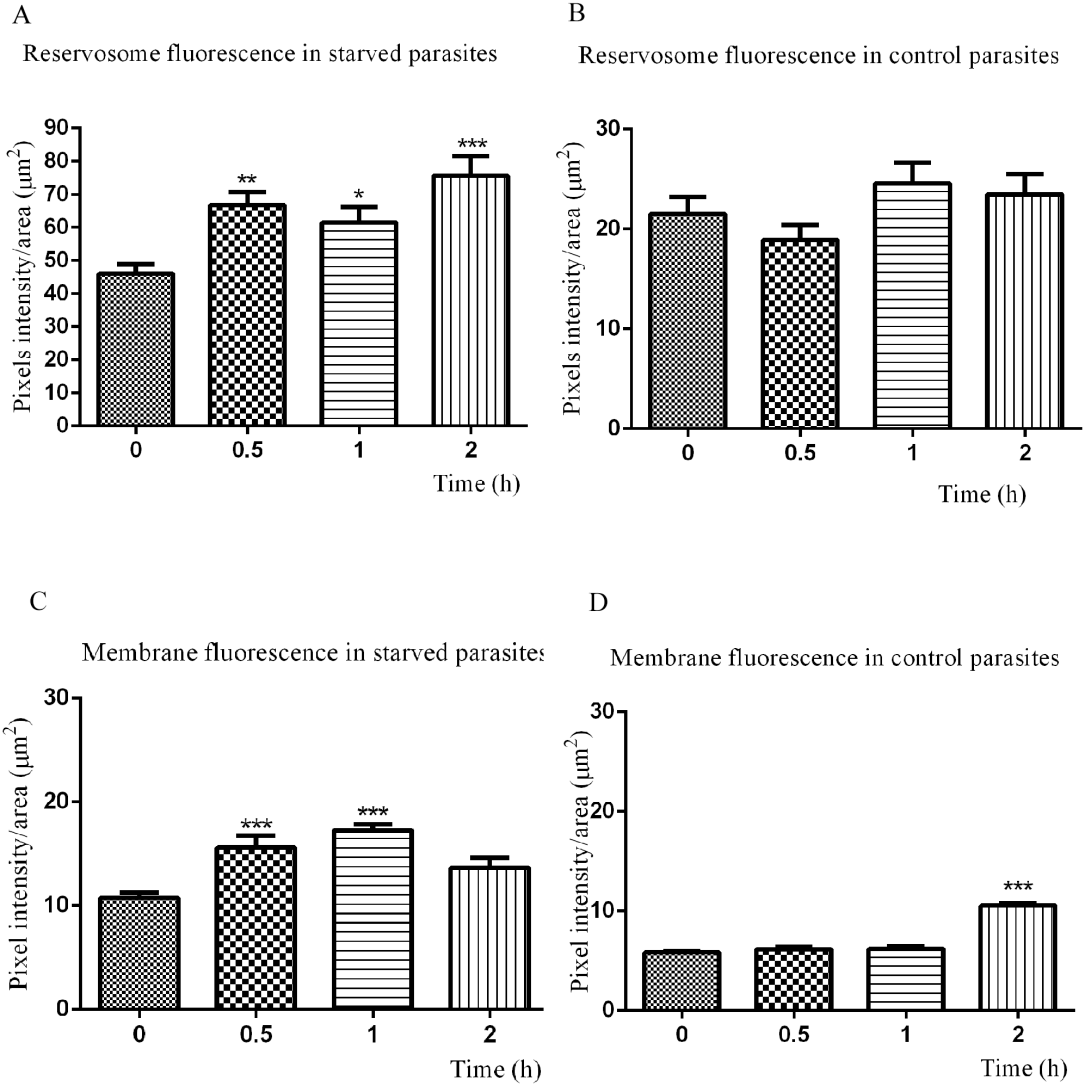
Quantification of NBD-cholesterol distribution in epimastigotes according to time. Starved parasites (A) uptake more LDL-NBD-cholesterol than controls (B), comparing fluorescence intensity in reservosomes/vesicles at time zero (immediately after washing off the tracer). The fluorescence intensity in reservosomes varies with time in starved parasites, but not significantly in control reservosomes, except after 2h. Starved parasites quickly redistribute the exogenous NBD-chol to their membranes (C) after 30 min, while in control cells (D) the NBD-chol rate increases only after 2 h of traffic. The results are expressed as the mean (±SD) and analysed with the one-way ANOVA test in comparison to time zero (*/**/*** significantly different, *P* < 0.05).

Although previous studies have noted the presence of cholesterol in the parasite plasma membrane [26–28], the sterol traffic in *T*. *cruzi* epimastigotes has not been investigated so far. We therefore decided to follow the uptake of fluorescent LDL-cholesterol in starved and control parasites. For this purpose, we incubated the parasites with LDL-NBD-cholesterol for 30 minutes to load reservosomes [4], washed excess tracer away from the medium and followed the NDB-cholesterol distribution from reservosomes to the rest of the cell at different times (Fig 2). Although fluorescence microscopy has no resolution to distinguish reservosomes from other compartments, the incorporation of the tracer in LDL particles that are taken in by epimastigotes only by endocytosis with a known kinetics, ensured that NBD-cholesterol were concentrated in reservosomes. Observing NBD fluorescence images captured under the same conditions, it was evident that lipid-starved parasites had taken up more cholesterol and started its distribution earlier than control parasites. For comparison, we quantified pixel fluorescence intensity in reservosomes and vesicles at the posterior region and confirmed that NBD-cholesterol initial endocytosis was almost two-fold higher in starved than control parasites (time zero in Fig 3A and 3B). There was already a small percentage of the fluorescent tracer in the membranes of both starved and control parasites (time zero in Fig 3C and 3D), which may be the result of the rapid redistribution from reservosomes to plasma membrane, especially in starved parasites.

After 30 minutes, 45% greater NBD-Chol fluorescence intensity was observed in the reservosomes and vesicles at the posterior region of starved parasites, indicating that reminiscent LDL-NBD-chol in cytostome and early endosomes reached the end of endocytic pathway [29]. After 1 h and 2 h of traffic, the fluorescence increase was 33.47% and 64.27%, respectively (Fig 3A). When we compared the initial NBD-chol fluorescence in the plasma membrane of starved parasites with that measured after 30 minutes, we found an increase of 44.79%, followed by 60.20% after 1 h of distribution throughout the cell (Fig 3C). After 2 h, a slight decay was observed, corresponding to 21% in relation to 1 h, most likely as a consequence of membrane traffic dynamics, which involves the balance between endocytic and secretory pathways. The reduction in plasma membrane fluorescence after 2 h was accompanied by the increase in reservosome content at the same time (Fig 3A), suggesting membrane traffic in the endocytic direction. Starved parasites distributed the NBD-Chol to plasma membrane faster because there was no notable difference until 1 h of traffic in control parasites (Fig 3D), reaching 76% of increase only after 2 h (Fig 3C).

### Epimastigotes present an acyl-CoA-cholesterol-acyltransferase—related enzyme

**Fig 4:**
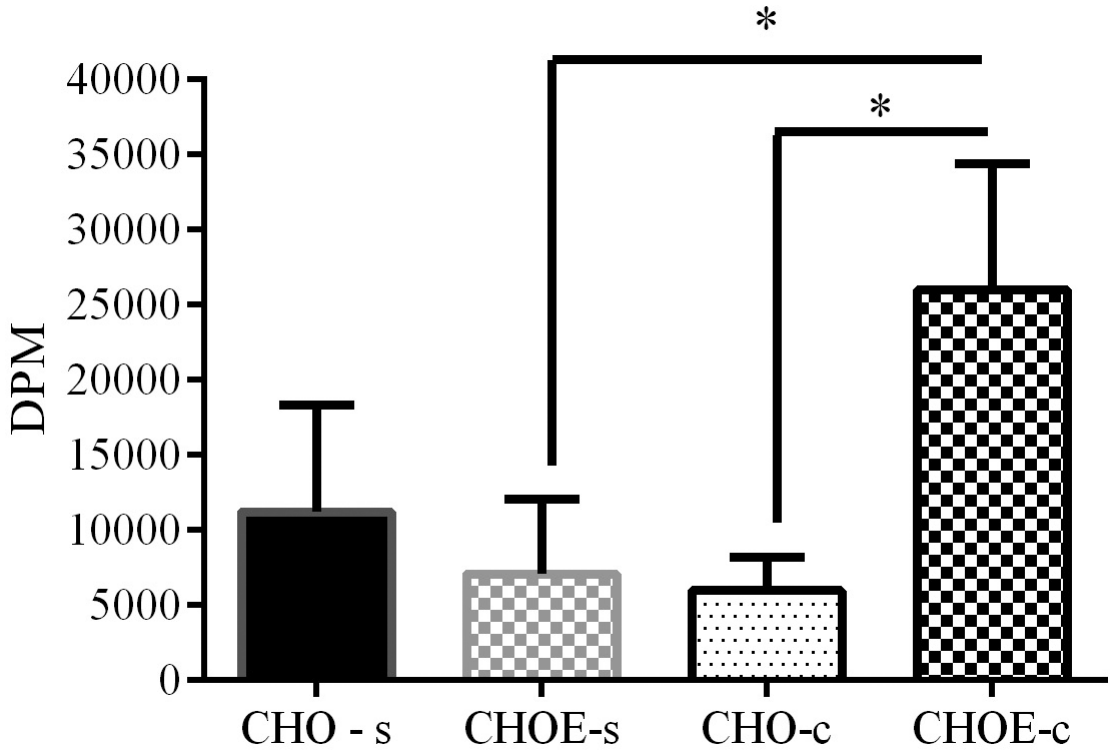
Incorporation of ^3^H-cholesterol into cholesteryl esters in epimastigotes. Parasites were incubated in LIT medium with 10% FCS (control) or 10% dFCS (starved) in the presence of LDL-^3^H-cholesterol (1,000,000 DPM; 2 mg/mL) for 3 days at 28°C. Parasites were washed and their lipids were extracted and analysed by TLC. The cholesterol (CHO) and cholesteryl-ester (CHOE) spots were scraped and the lipid eluted from silica. The lipid-associated radioactivity was measured by liquid scintillation counting. The results are expressed as the mean (±SD) per 1 mg of protein of three independent experiments analysed by one-way ANOVA followed by the Bonferroni test (*P<0.05).

In order to investigate the ability of epimastigotes to esterify cholesterol, the parasites were maintained in LIT medium plus 10% FCS or dFCS, supplemented with ^3^H-cholesterol-LDL for 3 days at 28°C. Fig 4 shows that control parasites esterified four-fold more cholesterol than starved epimastigotes. The lower rate of cholesterol esterification in starved parasites highlights the fact that these parasites, when deprived of lipids for a long time, used the previous stock of exogenous lipids in their immediate metabolism, most likely to restore and maintain membrane production for proliferation. The modulation of the production of cholesteryl esters by cholesterol availability is similar to that found in mammalian cells [30], with the important difference being that the endocytic uptake is the only cholesterol source.

**Fig 5:**
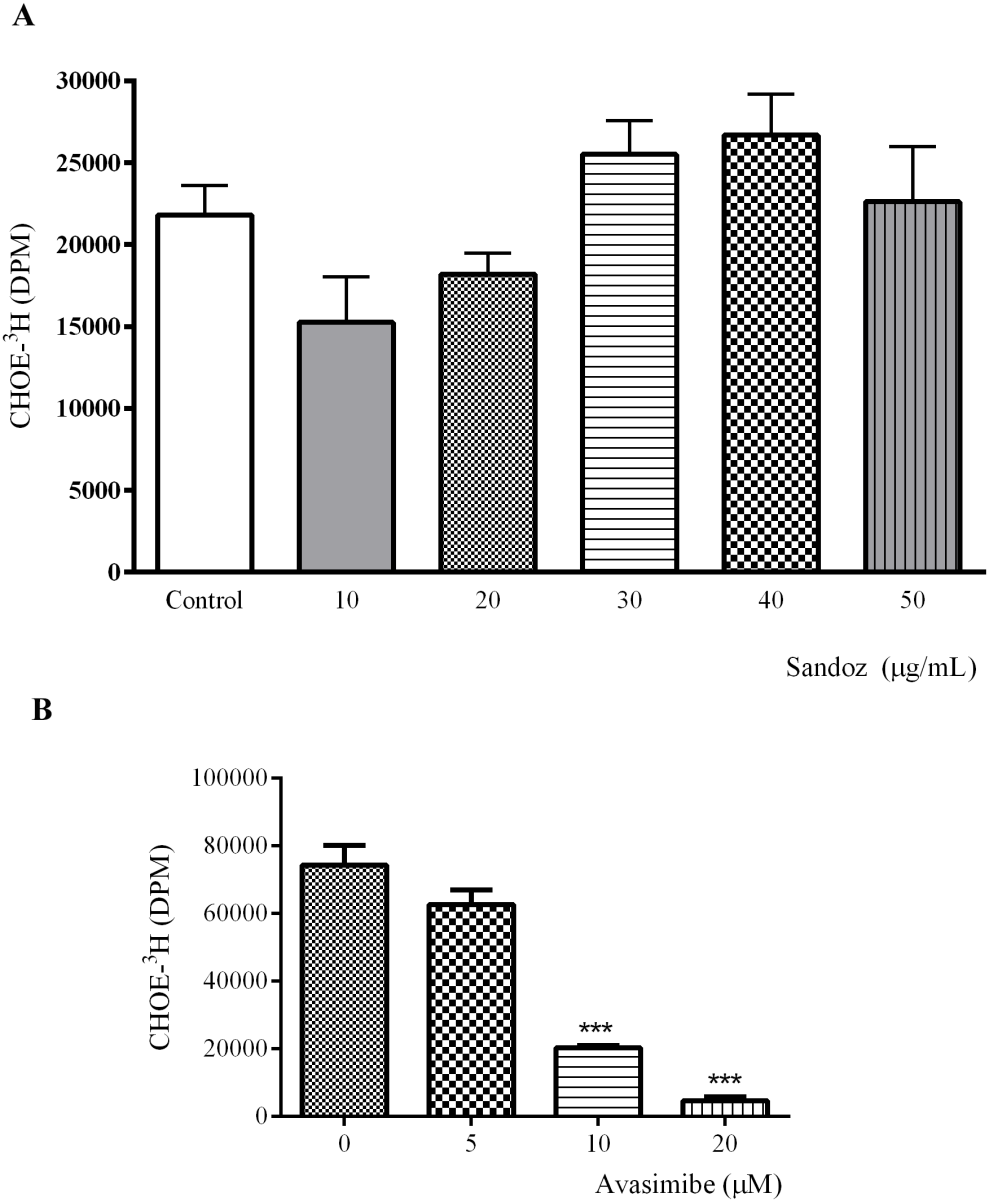
ACAT-related activity assay. The ability of *T*. *cruzi* epimastigotes to esterify cholesterol was tested using Sandoz 58-035-58-035 for 16–18 h (A) or Avasimibe for 24 h (B) in different concentrations, followed by addition of BSA-^3^H-palmitate (800,000 DPM; 1 mg/mL) for 24 h. Lipid analysis was performed by TLC; CHOE spots were scraped and the lipid eluted from silica. The lipid-associated radioactivity was measured by liquid scintillation counting. The results are expressed as the mean (±SD) per 1 mg of protein of two independent experiments in duplicate, and analysed by one-way ANOVA followed by the Bonferroni test (*P<0.05).

With these experiments, we have directly proven that *T*. *cruzi* epimastigotes esterify cholesterol, exhibiting an enzymatic activity similar to acyl-CoA-cholesterol-acyltransferase (ACAT). To further investigate this ACAT-like activity, we decided to test its susceptibility to two different non–specific ACAT inhibitors, Sandoz 58–035 58–035 and Avasimibe, at different concentrations. Cholesterol esterification was not affected by Sandoz 58–035 (Fig 5A), most likely because the parasite enzyme is different from ACAT described in higher eukaryotes. In contrast, we observed a drastic reduction of cholesteryl ester production after Avasimibe treatment in a dose-dependent manner (Fig 5B). Parasite viability during treatment with both inhibitors was monitored by two different tests, MTS and membrane permeability to propidium iodide. The parasites were not affected by the drugs (S1 Fig). Cholesterol loaded parasites, treated or not with Avasimibe were also examined under the electron microscope and compared with untreated parasites. We did not find any ultrastructural modification (S2 Fig).

In mammals, cholesteryl ester synthesis depends on the activity of two acyl-CoA: cholesterol-acyltransferases, with both enzymes localised in the endoplasmic reticulum but with different tissue distributions. Whereas ACAT-1 is ubiquitous and related to the formation of lipid bodies, ACAT-2 is restricted to intestines and the liver and acts on cholesterol absorption and LDL particle formation (reviewed in [30]). Sandoz 58–035 is capable of inhibiting ACAT activity and impairing cholesterol esterification in rat hepatoma cells, arterial smooth muscle, fibroblasts, human monocyte-derived macrophages, resulting an increasing free cholesterol efflux or reducing the acLDL binding and uptake to overcome the excess of toxic cholesterol [3, 25, 31]. Avasimibe is other non-selective ACAT inhibitor also involved in lowering cholesterol levels in cells. Rodriguez e Usher [32] showed similar results of this drug in lowering cholesterol levels as described for Sandoz 58–035. The authors also proposed that Avasimibe could directly alter scavenger receptor (SR) function or number. Additionally, Bemlih and co-workers [11] suggested that Avasimibe could reduce ACAT-1 expression and cholesterol ester synthesis in glioma cell lines. In this work, we did not explore the potential mechanisms involved in the esterification of sterols in *T*. *cruzi* and the susceptibility of its ACAT-like enzyme to different sterol substrates and other inhibitors. Since Sandoz did not produce effects on the parasite enzyme, we suggest that TcACAT might be structurally different from those described in higher eukaryotes.

In parasitic protozoa, it has been suggested that the presence of an acyl-CoA:cholesterol acyltransferase (ACAT) in *T*. *brucei* is responsible for the esterification of cholesterol from LDL and its subsequent accumulation in lipid bodies [33]. Subsequently, ACAT-related enzymes important for parasite growth and survival were characterised in *Toxoplasma gondii* [34, 35]. De Cicco *et al*. [17] demonstrated that *L*. *amazonensis* also esterifies cholesterol using ^3^H-palmitate complexed to BSA. However, an ACAT-related enzyme had not yet been identified and characterised. Recently, an elegant work using a bioinformatics approach to search for the missing enzymes of the *T*. *cruzi* sterol biosynthesis pathway, identified an ortholog of the *Saccharomyces cerevisiae* ERG10 gene, which codes for the yeast ACAT. The gene, called TcACAT, corresponds to a hypothetical protein. [36]. The authors also investigated the genetic diversity of this pathway within trypanosomatids and, surprisingly, did not find any gene corresponding to ERG10 in *T*. *brucei* or *Leishmania*.

For the first time the cholesterol from LDL particles was used to study cholesterol traffic and the ability of *T*. *cruzi* epimastigotes to insert cholesterol into their membranes. In addition, these results also sustain the hypothesis that epimastigotes use exogenous sterols as the first choice in situations of lipid nutritional stress, in an attempt to restore sterol levels and maintain parasite proliferation and metabolism. However, is not clear how cholesterol leaves reservosomes. Proteomic analysis of a reservosome fraction failed to detect the NPC1 and/or NPC2 proteins, but revealed a set of enzymes related to lipid metabolism, including lipases [7]. Furthermore, Torres *et al*. [8] described the presence of the TcABCA1 transporter in plasma membranes, flagellar pockets and reservosomes. The presence of this cholesterol transporter was further confirmed by our group after reservosome proteomic analysis. By some mechanism, cholesterol may emigrate from reservosomes using the TcABCA1.

Avasimibe has been considered a potent ACAT-1 inhibitor [11]. Its effect on the esterifying enzyme we identified in this work supports the hypothesis of a function similar to ACAT-1: namely, the formation of lipid bodies. *T*. *cruzi* is a cholesterol auxotrophic parasite that must scavenge this lipid from the host. The capacity to store cholesterol efficiently and via multiple pathways is surely important for this parasite. We had previously demonstrated that epimastigotes store cholesterol and cholesteryl ester inside reservosomes [5] and now our data show that the mobilisation of this stock is directed towards more than one fate: in starved parasites, the priority is the insertion of cholesterol into membranes to maintain parasite proliferation, whereas in normal conditions, esterification predominates, most likely to remove the excess of free cholesterol, leading the formation of lipid bodies. This hypothesis implies in a unidirectional traffic of cholesterol from reservosomes to lipid bodies. However, further experiments are necessary to determine if the site of cholesterol esterification is the endoplasmic reticulum, as in mammal cells. In this case, endocytosed cholesterol molecules would leave reservosomes, be modified in the endoplasmic reticulum and participate in the formation of a new lipid body or be transferred to a pre-existing one. We currently do not know to where cholesteryl esters are addressed, and if parasites perform cholesterol efflux.

In summary, these results emphasise the importance of cholesterol for epimastigote metabolism. The ability to insert it into parasite membranes or use it as alternative sterol lipid, mostly in situations of lipid nutritional stress, reveal an excellent mechanism of parasite adaptation. Moreover, the detection of an ACAT-related enzyme implies that exogenous cholesterol has a role in lipid reservoirs in epimastigotes.

## Supporting Information

S1 Fig Parasites viability during ACAT inhibitors treatment. The viability of epimastigotes treated with Sandoz for 40h or Avasimibe for 48h, at the indicated concentrations, was evaluated using MTS (A and B) or permeability to propidium iodide (C). Untreated parasites were considered 100% viable in A and B. Parasites treated with the solvent (DMSO) were also evaluated. The results are expressed as the mean (±SD) of two independent experiments in duplicate.
(TIF)

S2 Fig Ultrastructure of Avasimibe treated epimastigotes. Cholesterol loaded epimastigotes, treated or not with 20 μM Avasimibe for 48h, were processed for transmission electron microscopy. We did not find any differences between membranes and organelles from untreated (A) and treated parasites (B—F). (B) Longitudinal section of a epimastigote presenting intact nucleus, kinetoplast, reservosomes, plasma membrane and flagellar pocket. (C) Transversal section showing reservosomes with lipid inclusions. Endoplasmic reticulum is closely associated with reservosomes. (D) Longitudinal section, with reservosomes and lipid droplets at the posterior end of the parasite. (E) Endoplasmic reticulum surrounding a lipid droplet adjacent to the nucleus. (F) Many lipid droplets and reservosomes. N—nucleus; K—Kinetoplast; FP—flagellar pocket; L—lipid droplet; R—reservosome; Cy—cytostome; F—flagellum; ER—endoplasmic reticulum. Bars correspond to 0.25 μm.
(TIF)
