# Supplementary figures and images for: Trypanosoma cruzi Epimastigotes Are Able to Manage Internal Cholesterol Levels under Nutritional Lipid Stress Conditions
Source: PLoS One. 2015 Jun 11;10(6):e0128949. doi: 10.1371/journal.pone.0128949 (PMC4466137; doi:10.1371/journal.pone.0128949)

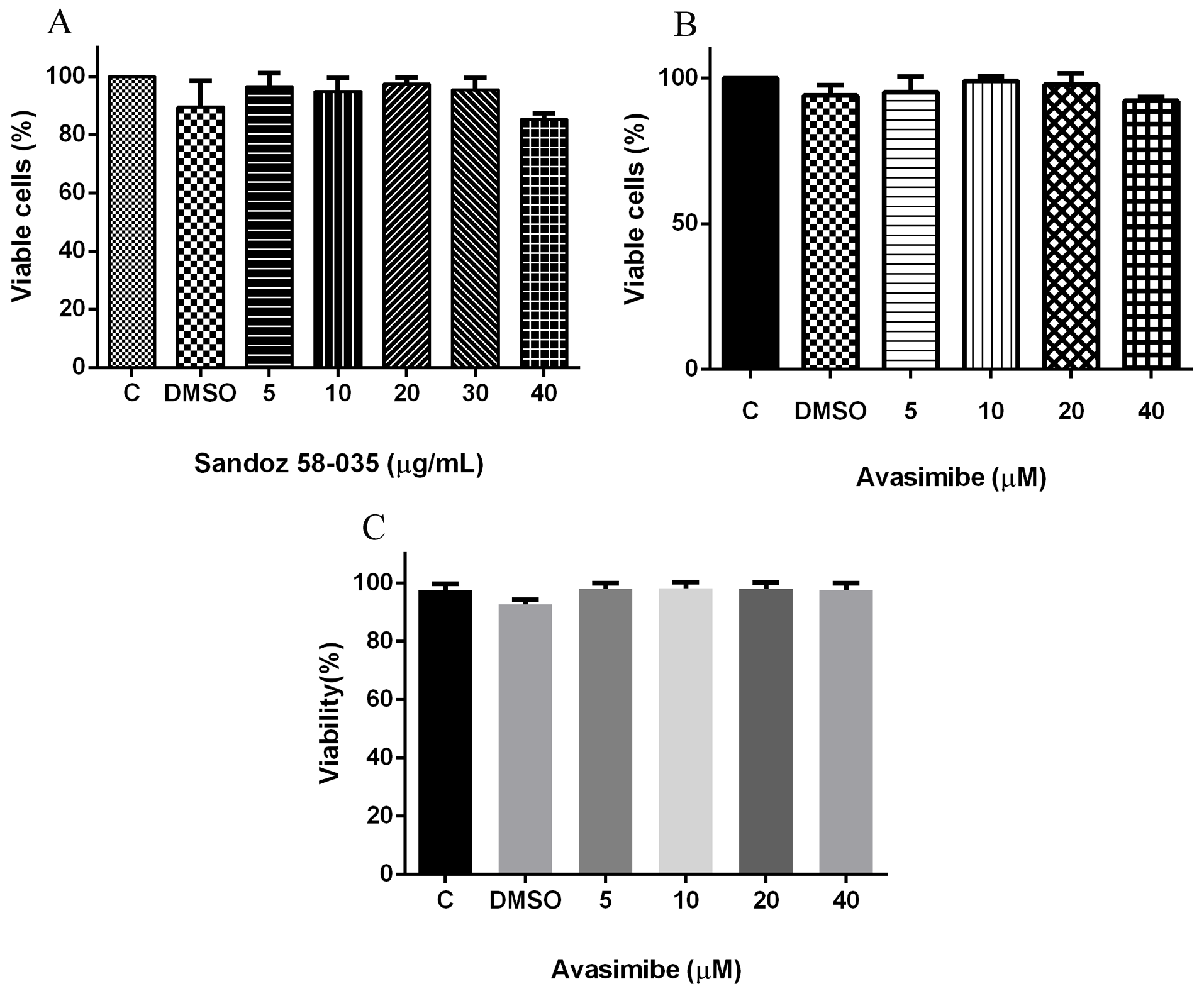

Supplement: S1 Fig — The viability of epimastigotes treated with Sandoz for 40h or Avasimibe for 48h, at the indicated concentrations, was evaluated using MTS (A and B) or permeability to propidium iodide (C). Untreated parasites were considered 100% viable in A and B. Parasites treated with the solvent (DMSO) were also evaluated. The results are expressed as the mean (±SD) of two independent experiments in duplicate. (TIF) [file pone.0128949.s001.tif]

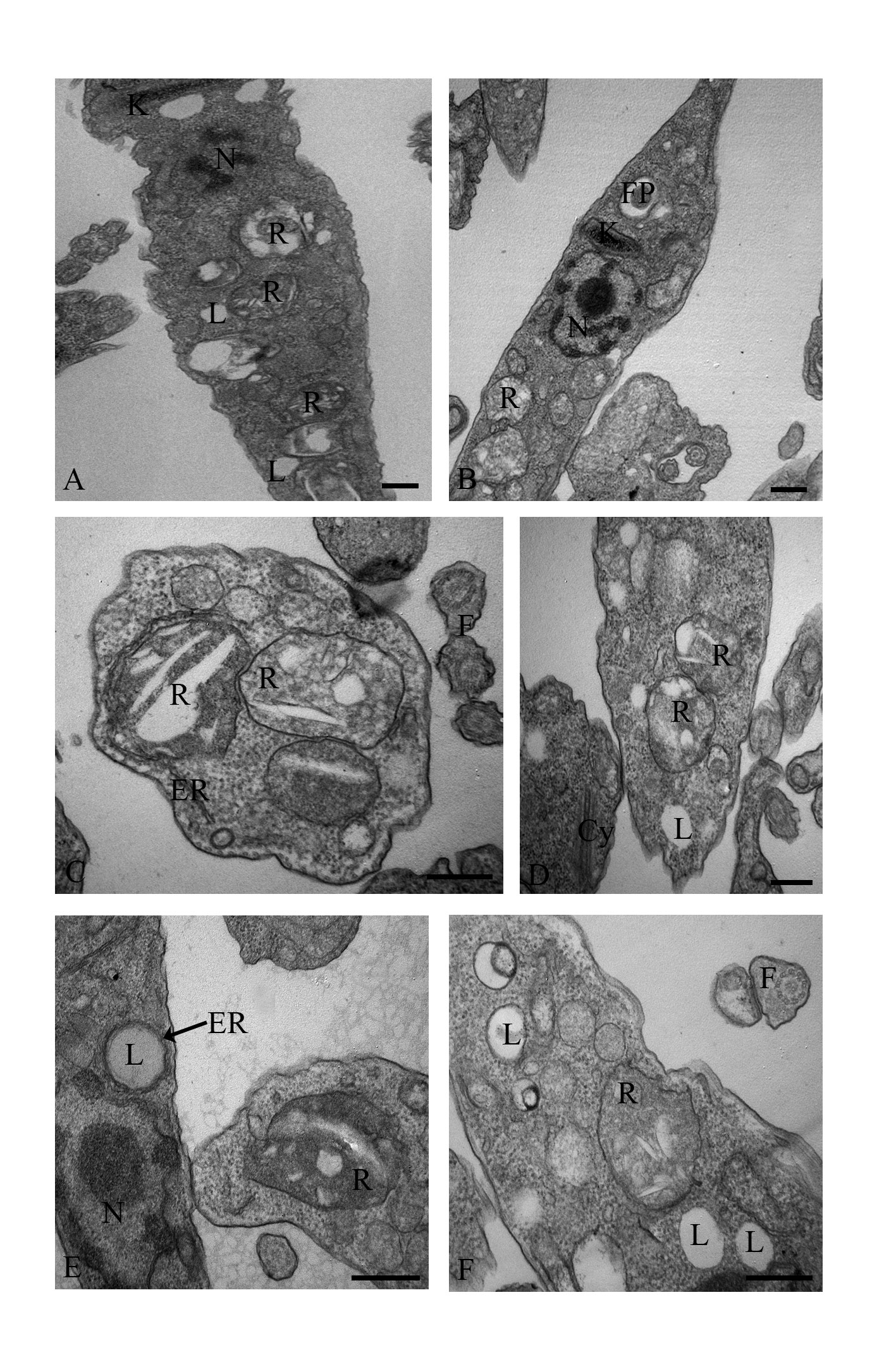

Supplement: S2 Fig — Cholesterol loaded epimastigotes, treated or not with 20 μM Avasimibe for 48h, were processed for transmission electron microscopy. We did not find any differences between membranes and organelles from untreated (A) and treated parasites (B—F). (B) Longitudinal section of a epimastigote presenting intact nucleus, kinetoplast, reservosomes, plasma membrane and flagellar pocket. (C) Transversal section showing reservosomes with lipid inclusions. Endoplasmic reticulum is closely associated with reservosomes. (D) Longitudinal section, with reservosomes and lipid droplets at the posterior end of the parasite. (E) Endoplasmic reticulum surrounding a lipid droplet adjacent to the nucleus. (F) Many lipid droplets and reservosomes. N—nucleus; K—Kinetoplast; FP—flagellar pocket; L—lipid droplet; R—reservosome; Cy—cytostome; F—flagellum; ER—endoplasmic reticulum. Bars correspond to 0.25 μm. (TIF) [file pone.0128949.s002.tif]
